# Supplementary material for: Adolescent screen time and unhealthy food consumption in the context of the digital development in New Caledonia
Source: PLoS One. 2023 May 8;18(5):e0285374. doi: 10.1371/journal.pone.0285374 (PMC10166542; doi:10.1371/journal.pone.0285374)
Supplement: Attachment — Submitted filename: Reviewer. Adolescent screen time and unhealthy food.ANG.docx [file pone.0285374.s001.docx]

**RESPONSES TO EDITORIAL OFFICE AND REVIEWERS**

**MANUSCRIPT PONE-D-21-27771**

**Adolescent screen time and unhealthy food consumption**

**in the context of the digital development in New Caledonia**

*Akila Nedjar-Guerre, Guillaume Wattelez, Christophe Serra-Mallol, Stéphane Frayon, Olivier Galy*

**Authors:** We thank the reviewers for their appraisals. We have done our best to address the concerns and hope that the manuscript is now suitable for publication.

COMMENTS FROM EDITORIAL OFFICE:

**Reviewer #1**

Reviewer #1: (1) If the paper is considered unsuitable for publication in its present form, does the study itself show sufficient potential that the authors should be encouraged to resubmit a revised version?

The paper is unsuitable in its current form, but given it is still relatively rare to see research by scholars of the Pacific islands, from an institution in the Pacific islands, about the Pacific islands (awesome!), it definitely merits revision and resubmission. The main points to address are outlined in the remainder of the review.

(2) What are the main claims of the paper and how significant are they for the discipline?

The paper identifies correlations between screen time and unhealthy food consumption amongst adolescents in New Caledonia. The finding itself is not particularly new or significant generally, because links between screen time and unhealthy food consumption in many parts of the world are well-established (e.g. Alex Brewis et al 2011 ‘Body norms and fat stigma’, Anne Becker 2005 ‘Television, disordered eating…’), and highlight that the content of the screen time is as important for influencing diet as the time. The same global structural inequalities underpin both; in addition, significant amounts of food marketing can occur via screens (e.g. in-game product placement, advertising, etc) and this space is largely unregulated. However, it is new to get this sort of research carried out in the islands of the Pacific by local researchers, and for this reason it should be commended.

**Q1 Overall, the conclusion that monitoring of screen use is necessary lacks nuance, and it is also hard to see how this recommendation has come from the data collected. Likewise, the conclusion that dietary or health education is necessary also lacks nuance, and it is likewise hard to see how this recommendation came from the data collected. The paper would be richer engaging with some of the critiques of these to recommend new ways forward, rather than distilling recommendations to education and surveillance (the methods we have employed for the past 100 years with no really evident change - see McLennan et al 2018 'The problem with...'). Or, if these are the best ways forward, giving more evidence that they come from the data and are likely to have an impact would be useful.**

**R1. You are right, the data do not allow us to give health recommendations. So, we have removed this information in the conclusion. Please see page 25; line 417-418**

**Q2.Are the claims properly placed in the context of the previous literature ? Have the authors treated the literature fairly?**

Some literature is treated really well. Other literature is not considered, and would strengthen the manuscript. In particular:

- Links between screens and unhealthy food (e.g. Maricarmen Vizcaino 2020 ‘From TVs to tablets’), and the importance of the screen content to shaping food behaviours (e.g. Brewis and Becker above)

**R2. These references are interesting and we have incorporated them into the text. See lines 360-362, p.22**

**Q3. A critical approach to recommending health education or intervention, rather than addressing structural factors that lead to poor health, to address unhealthy food consumption in the UK (Ulijaszek and McLennan 2016 ‘Framing obesity in UK policy…’), Pacific (McLennan et al 2018, see above) or elsewhere.**

**R3. Thank you for your comments and references. The question of recommendations is not dealt with very much because it is primarily a question of seeing the links between screens and poor nutrition, which has never been dealt with in the territory.**

**Q4. Literature on ‘screen time’ as TV time in the Pacific islands. The authors claim that the literature is sparse, but it would be useful to know what specifically has been understudied (e.g. television, computers, internet, other things?)**

**R4 To our knowledge, this is the first study about the links between technological equipment, screen time and food consumption in New Caledonia. While a study has been conducted in Vanuatu on technology and obesity (Sun et al, 2017), or other studies in Australia (Maher et al, 2012), they do not address questions about community differences (European, Polynesian, Melanesian adolescents), and socio-economic criteria in a multicultural territory, which has a big impact on this vulnerable population.**

**Q5.Some claims are a little loose and require more accuracy and/or justification and explanation throughout the paper. For example:**

**“The digital transition has been accompanied by a socioeconomic transition” – what sort of socioeconomic transition does the author mean specifically, and what is their evidence for this?**

**R.5. We have taken your question into account. The socio-economic transition has been explained in the background of the article. Please see, p. 2-3, line 39-62**

**Q6. Or, if they just mean that “Increasing connectivity of islands to the internet has led to changes in the way people engage with screens” then say this more specifically and give evidence to back up this particular point (so, if they are claiming there has been an increase in screen use, there needs to be evidence of ‘before’ and ‘now’, not just a single data point from 2018). If the authors are going to mention things like a ‘socioeconomic transition’ they must engage with what they mean by this specifically; personally I’m not sure this particular term is needed in this paper.**

**R6 : Indeed, the term "digital development" is more appropriate than digital transition because we have not studied the effects of the transition on the economic and social aspects of the inhabitants. The term digital transition has been replaced by digital development throughout the article.**

**Similarly, we have made greater reference to the 2011 study. See p. 6 line 115-116**

**In the background section we have included graphs on the evolution of connectivity since 2009 in the different areas studied.**

**Q7. It would be useful if the authors could explain in the introduction what they mean by ‘screen time’ and/or ‘screens’. Their text implies they are talking about use of internet-connected devices, but ‘screen time’ in the past has also been used to mean television time, and there is a vast literature on this topic already. Some of the data they cite relates to the former, and some to the latter, and this needs untangling and clarification. Does ‘screen time’ mean the same thing in New Caledonia as it does in other contexts? For example, some children engaged in remote learning will spend large amounts of time at a screen, but this is very different to, say, watching commercial television or playing games (that feature advertising or not) or talking to loved ones. In this context, not all screen time may be bad – although the paper implies that this is the case. And, what is the difference between using a phone and a computer/tablet? Are there different activities? Do they serve different purposes? What about on weekends versus weekdays? What sort of screen use could be encouraged versus not? There are also a lot of positives from being connected, presumably? Or maybe not?**

**R7 : Screen time was used as a factor that explains consumption of unhealthy food and beverages, depending on the different types of screens. We do not discuss what young adolescents do in front of their screens and whether it is good or bad. We indicate this within the limits of the discussion. See p. 22 line 406-409**

**Q8 It would be useful if the authors could engage critically with the notion of the ‘digital transition’. The evidence they cite indicates that growing internet connectivity has changed people’s use of technology (specifically screens, in this paper), and that the changes have been different in different contexts. This is indicative not of some sort of universal and inevitable ‘transition’ but instead context-specific changes in the ways people live, work and play. Indeed, the notion of transitions has been critiqued thoroughly in the Pacific islands context, most notably around the idea of the ‘nutrition transition’. Do they really mean increased internet connectivity of people living in the Pacific islands? If so, they could just state this rather than using the problematic notion of ‘digital transition’.**

**R8 : Yes, indeed, there is an increase connectivity among the Caledonian population. We therefore use the term digital development in the territory instead of digital transition (see answer 6)**

**Q9 The statement that New Caledonian adolescents might reach the same numbers as those in France is particularly problematic in this regard, as it reinforces colonial notions that islanders all inevitably want to ‘develop’ to become more like their colonisers.**

**R9 : We only want to compare with another country that is very advanced in digital connectivity. With this comparison, we do not induce that New Caledonian population should reach a specific level of France. That is why, to preserve readers of this perception, we also compare with Australia. Please see p 19. line 328-330**

**Q10 The authors should also be mindful that division into ‘Melanesian’ or ‘Polynesian’ is also a colonial construct – it would be useful if they could explain whether they are replicating the colonial category (and if so, why), or whether these categories are now used by the people of New Caledonia to describe themselves.**

**R10 : New Caledonia is a multicultural country with different influences from Pacific and Europe. Although it is a colonial construction (Tcherkezoff, 2009), these categories are largely used in New Caledonia, mainly for census that is done each 5 years (ISEE-NC, 2019). Moreover, the tribal way of life in New Caledonia only concerns Melanesian peoples (not Polynesians). These categories have been used by the populations themselves, who have appropriated them, including the Kanaks who define themselves as Melanesian indigenous populations.**

**Q11 There are a number of other concepts where it would be of value if the author could engage critically with them rather than just applying Western notions to making sense of islander lives. For example:**

**- The notion of ‘sedentary time’ (as something to be avoided) is not necessarily useful in a Pacific islands context where sitting together is an important social practice. Not sure what the context is in NC though.**

**R11 : When we measure sedentary time, we measure it from a physiological point of view by accelerometers to assess physical activity (not realised in this work). So, we replaced sedentary time with physical inactivity in p.8, line 140.**

**Q12 Obesity has been demonstrated as related to much more than just diet, place of living, activity, or other individual behaviours. Indeed, the focus on individual behaviours has also been critiqued in some of the sources cited by the authors.**

**R12 : You’re right, obesity is linked to invidual, interindividual and socio economical aspects that need to be considered. In this work we focused of efforts screen time variable that may indirectly influence obesity. So, in the revised manuscript we focused or message mainly on this variable.**

Q13 Finally, there is discussion about rural activities and rural v urban; more needs to be said about these and what they mean for the research in the introduction. A map would be useful.

**R13 : Please see p. 8, lines 142-144. A map has been added to the background on rural and urban areas in New Caledonia.**

(4) Do the data and analyses fully support the claims? If not, what other evidence is required?

**Q14 There is some confusion in the final conclusion – if I have read it correctly, the authors are right to highlight correlation between screen time and unhealthy food. However, their claim at the start of the paper – that unhealthy food consumption increases with screen time (can they show this causation?), or that health education programs are the answer, are less robust. There is plenty of literature illustrating the failure of health education programs to address the structural inequities underpinning unhealthy food consumption. There is also literature pointing out how recommending health education programs deflects blame from food companies. That the authors make this recommendation is especially problematic given the paper is funded by Nestle. Overall, this recommendation requires revision and/or considerably more justification if the paper is to be published. In addition, the authors need to critically examine the funding source, and how it can influence the sorts of research being done (or not) in New Caledonia, how it might influence research framing and conclusions, especially given that the findings are in line with global food industry lobby narratives.**

**R14 : we are studying the links between screen time and poor diet but we can’t prove the nature of the causality. We add to the limitations the fact that questions about whether young people eat in front of their screens were not asked. See p. 22 line 399-401**

**We would like to underline that this funding was both funded by “Nestlé Fundation” and the University of New Caledonia as written in the Acknowledgment section. An independent scientific committee is nomited and “Nestlé Fundation” has no role in this study design, results and interpretation presented here and gave only a financial support to do this study against acknowledgements. Please see p.23, 427-429**

**Concerning the recommendations, the data do not allow conclusions to be drawn on health education recommendations. We have removed this statement from the article as described in answer 1**

**Q15. The graphs do not add much value, and they seem out of place in the methodology section. I wonder whether they would be best presented in prose or even in data table format? They also require explanation in the text: What can we conclude from each figure and how does each add to the argument? A map instead would be valuable.**

**R15. We have moved the graphs to the background section. This allows a better understanding of the evolution of digital connectivity in the rural and urban areas studied.** **In the background,** **we have added a map of rural and urban areas by number of inhabitants.

Q16. Data were collected prior to the COVID-19 pandemic. I think this is important to note, because the pandemic has had many effects on internet connectivity and screen use.**

**R16 The questionnaires were indeed filled before the covid-19 period. We have mentioned this information in the methodology p. 10, line 190-191 and in the conclusion by indicating that screen times might have increased after covid-19 (p.23, line 419-421).**

(5) PLOS ONE encourages authors to publish detailed protocols and algorithms as supporting information online. Do any particular methods used in the manuscript warrant such treatment? If a protocol is already provided, for example for a randomized controlled trial, are there any important deviations from it? If so, have the authors explained adequately why the deviations occurred?
N/A

(6) Are details of the methodology sufficient to allow the experiments to be reproduced?

To a certain extent yes, although bear in mind that societies change over time, so while the methods could be reproduced, it is unlikely they would yield the same results, or that the results would be interpreted in the same way.

**Q17. It would be useful if the authors could add some information on how the different people listed in the acknowledgements were involved in data collection and analysis. It is a large team in the acknowledgements, and huge coverage of the nation, and this coordination in itself is an important part of the methodology because it is not easy to do this in Pacific island nations. Kudos!**

**R17 In the “Author contribution” section, we have mentioned the names concerning the acknowledgements on each important stages: the creation of the questionnaire and the setting up of the field protocol, the data collection and cross-referencing of statistical data and the writing of the article.**

**Q18 The parental permission is also incredibly high - what led to this? Local connections? Reward? Another thing that would help others to reproduce some of the work should that be needed.**

**R18 Please see p 10, lines 195-198**

**Q19 How did the authors go from data to the conclusions they drew?**

**R19 The last part of the conclusion has been modified to fit the data. See the last paragraph of the conclusion.**

**Q. 20 What language/s was the survey delivered in, and how might this have affected results?**

**R.20 The language used is French because it is the common and official language of all the inhabitants of the territory. The questionnaires were conducted in schools that use French in their teaching. So, we assume that for young people, the use of the language has no impact on the results. Please consider p.10 line 200-201.**

**Q. 21 Is the European standard for determining ‘urban’ appropriate and why?**

**R 21: To determine urban areas, we used common tools of national census as follow and inserted in the revised manuscript please see p. 11 line 221-226 :**

**“Densely populated areas comprising at least 50 000 inhabitants in a continuous zone with more than 500 inhabitants/km2 were classified as urban. Areas with more than 50 000 inhabitants in a continuous area of over 100 inhabitants/km2 or areas adjacent to an urban area were classified as semiurban. Rural areas were defined as those areas that did not fulfill the conditions required to qualify as urban or semiurban”.**

**Q 22 Why only use responses on unhealthy foods in data analysis? This decision requires justification.**

**R22 Previous studies by the research team have shown links between unhealthy foods and obesity (ref. 21) in adolescents caledonian. So, in this work it was evident to move forward on this was and we focused our research on the links between screen time, drinks and unhealthy foods.**

**Q 23 Where did students carry out the survey? Did they imagine it was school homework? Was there potential for the setting to skew or influence their responses (e.g. to impress a teacher or ensure their parents don’t find out when they use their phone?)?**

**R23 : Students completed the surveys online in front of a screen (tablet or school computer) while in their classrooms. The study was supervised by researchers with the presence of teachers. Adolescents knew that data was anonymous and explained before the study by a member of the research team. Similarly, teachers were not involved in the protocol.**

**Q 24 It would be useful for the data collection instruments to be included as Addenda to the paper.**

**R 24. The questionnaire is added to the research article.** [**https://www.protocols.io/private/6E61F26F027411EDAB850A58A9FEAC02**](https://www.protocols.io/private/6E61F26F027411EDAB850A58A9FEAC02)

**Q 25 With whose ethics board was the study approved? Did you also need permission from schools? What were the potential harms (e.g. talking about food with adolescents can contribute to body shame or eating changes, see Brewis and Becker)?**

**R 25: The protocol has been approved by the Caledonian consultative committee (CCE 2018-06 001). The Vice-Chancelor gave his agreement, the principals from each schools, parents and then participants. See p.10, line 187-189.**

**The questionnaires were anonymous and we used likert sclaes, binar reponses, or numbers to choose according to questions. We used international quesitonnaires used and validated in adolsecnt population and whe possible adapted to communities. So, we assume that there were no embarrassment concerning questions adressed.**

(8) Is any software created by the authors freely available?
N/A

(9) Is the manuscript well organized and written clearly enough to be accessible to non-specialists?

Yes, overall it is well-written and easy to read, although there are a number of sweeping generalisations that are possibly too simple, and require additional nuance and/or accuracy.

(10) Is it your opinion that this manuscript contains an NIH-defined experiment of Dual Use concern?
N/A

**Reviewer #2**: This paper has been found of some interest in its field. Besides, the research question posed by the author is easily identifiable and recognized the original aspect. Therefore, the study design was carefully carried out.

However, only some questions emerge:

**Q1. explain better why the author didn’t explore the time spent on video games (line 345-346);**

**R1 : In this survey, we got information about time spent in front of computer and mobile phone. This time includes a part of video game time. Unfortunately, we did not get any information about video games specifically. However, we agree that time spent on video game consoles could be considered when studying screen time. We added this limitation p. 22 line 398-399.**

**Q2 why didn’t consider weight and body mass index in correlation to screen time and unhealthy food consumption? Please, add these data if available. In the other case insert this point in limitations**

**R2 : Thank you for this interesting comment. Many researches showed such correlations with BMI (references n° 3, 21 and 31). However, the current research focused on junk food consumption and screen time according to place of living, SES and ethnic community. That is why we did not attempt to identify correlations between BMI, junk food consumption and screen. Another future study could be dedicated to this issue.**

**Q3 Also discuss in Discussion Section other papers conducted in area with similar cultural diversity and gradients of urbanization and digital infrastructure development (for example, Camila Wohlgemuth Schaan, et al. Unhealthy snack intake modifies the association between screen-based sedentary time and metabolic syndrome in Brazilian adolescents. Int J Behav Nutr Phys Act. 2019; 16: 115; Silvia A. González, et al. Prevalence and Associated Factors of Excessive Recreational Screen Time Among Colombian Children and Adolescents. Int J Public Health. 2022; 67: 1604217).**

**R3 : Many thanks for these interesting references. I have added them p.20, line 357-359**
